# Is it time to drop the ‘knowledge translation’ metaphor? A critical literature review

**DOI:** 10.1258/jrsm.2011.110285

**Published:** 2011-12

**Authors:** Trisha Greenhalgh, Sietse Wieringa

**Affiliations:** 1 Global Health, Policy and Innovation Unit, Centre for Primary Care and Public Health, Barts and The London School of Medicine and Dentistry, London E1 2AB, UK; 2 HAWeb Implementation Unit, Nederlands Huisarts Genootschap/Landelijke Huisartsen Vereniging, Utrecht, The Netherlands

## Abstract

The literature on ‘knowledge translation’ presents challenges for the reviewer because different terms have been used to describe the generation, sharing and application of knowledge and different research approaches embrace different philosophical positions on what knowledge is. We present a narrative review of this literature which deliberately sought to highlight rather than resolve tensions between these different framings. Our findings suggest that while ‘translation’ is a widely used metaphor in medicine, it constrains how we conceptualise and study the link between knowledge and practice. The ‘translation’ metaphor has, arguably, led to particular difficulties in the fields of ‘evidence-based management’ and ‘evidence-based policymaking’ – where it seems that knowledge obstinately refuses to be driven unproblematically into practice. Many non-medical disciplines such as philosophy, sociology and organization science conceptualise knowledge very differently, as being (for example) ‘created’, ‘constructed’, ‘embodied’, ‘performed’ and ‘collectively negotiated’ – and also as being value-laden and tending to serve the vested interests of dominant élites. We propose that applying this wider range of metaphors and models would allow us to research the link between knowledge and practice in more creative and critical ways. We conclude that research should move beyond a narrow focus on the ‘know–do gap’ to cover a richer agenda, including: (a) the situation-specific practical wisdom (phronesis) that underpins clinical judgement; (b) the tacit knowledge that is built and shared among practitioners (‘mindlines’); (c) the complex links between power and knowledge; and (d) approaches to facilitating macro-level knowledge partnerships between researchers, practitioners, policymakers and commercial interests.

## Introduction

The first article indexed on Medline under ‘knowledge translation’ was published (in French) in 1972.^1^ It proposed what is now termed ‘T1′ or ‘bench to bedside’ knowledge translation – measures to ensure that laboratory discoveries would be applied in the diagnosis or treatment of disease. A second phase of knowledge translation – ‘T2’ or ‘campus to clinic’ – considers how findings from health services research (systematic reviews, randomized trials and so on, perhaps presented as clinical practice guidelines) could be more widely adopted in practice and policy.

The past five years have seen unprecedented investment in knowledge translation research. In 2006, the US National Institute of Health introduced its Clinical and Translational Science Award (CTSA) program with the goal of funding 60 centres by 2012, at an annual cost of US$500 million.^2^ In the same year, the UK Cooksey Report set out an ambitious strategy for translational research in basic and clinical sciences.^3^ This led to the establishment of the Office for Strategic Coordination of Health Research, with ‘translational medicine’ (overseen by a dedicated Translational Medicine Board) featuring prominently in its £1.7 billion annual budget. Of 27 journals containing the word ‘translational’ listed on the NCBI index of medical journals, 18 have been launched since 2008.

As the T1/T2 taxonomy suggests, the terms ‘knowledge translation’ and ‘translational medicine’ are associated with two separate processes – (a) incorporation of basic science innovations into the design of new tests and treatments, and (b) uptake of validated tests and treatments into clinical practice (e.g. via evidence-based guidelines) and policy-making (e.g. via service level agreements and incentive structures). The former tends to be viewed largely as a scientific and technical process and the latter as incorporating behavioural, organizational and perhaps political elements.^2^ In this paper, we argue that in a number of settings – the clinical encounter, organization and management of healthcare, and the policy-making process (including how research priorities are set) – the metaphor ‘knowledge translation’ constrains thinking. Drawing mainly on disciplines outside medicine, we introduce a range of alternative metaphors and models which highlight the fundamentally social ways in which knowledge emerges, circulates and gets applied in practice. We conclude by suggesting that much could be gained by applying these metaphors and models more widely in the domain of medicine and healthcare.

## Search strategy and method

We were already aware of systematic reviews,^4–6^ overviews and concept maps;^2,7–14^ and academic papers^15–17^ on knowledge translation. We pursued papers from the reference lists of these sources and identified more recent articles by citation-tracking them in Google Scholar. We also had a database of sources on this topic which TG had begun to collect opportunistically in 2004. We summarized and drew together findings from these diverse and conflicting sources using narrative synthesis.

## Knowledge translation: unpacking the metaphor

Knowledge translation was defined at a consensus meeting of the World Health Organization in 2005 as ‘the synthesis, exchange and application of knowledge by relevant stakeholders to accelerate the benefits of global and local innovation in strengthening health systems and advancing people's health’ (page 2).^12^ Successful knowledge translation (implicitly, T2) was conceptualised as dependent on ‘supply’ or ‘push factors’ (availability of evidence; appropriate packaging, e.g. in ‘evidence-based actionable messages’; credible knowledge brokers and opinion leaders); and ‘demand’ or ‘pull factors’ (e.g. local knowledge champions; political support for implementation of particular research evidence; strategic presence on local decision-making bodies). Barriers to knowledge translation were likewise divided into push factors (e.g. evidence too complex; cost of producing, packaging and distributing evidence too prohibitive; poor local access to relevant evidence) and pull factors (e.g. low demand for scientific evidence by policymakers; political and/or financial reasons for not acting on evidence; ‘paradigm differences’ between researchers, policymakers and practitioners).^12^

Many published analyses of the knowledge translation challenge offer similar taxonomies of problems and solutions. Clinicians, it is lamented, only rarely follow evidence-based guidelines; managers and policymakers fail to draw consistently on robust evidence when designing services or allocating resources. Solutions to these problems are framed in terms of a more efficient ‘evidence pathway’,^7^ ‘evidence-based decision support’,^14^ ‘evidence-based policymaking’^15^ and ‘evidence-based management’^16,17^ – all of which entail the controlled supply of research evidence that has been vetted, summarized and made accessible to its intended audience and/or the shaping of demand for this evidence through education, facilitation, financial incentives or inscribing decision pathways into technology.

Three assumptions underpin the knowledge translation metaphor. The first is that ‘knowledge’ equates with objective, impersonal research findings – a form of what Aristotle called *episteme* and later writers have called explicit knowledge. In basic science, research evidence means consistent and reproducible laboratory findings;^2,18^ in health services research, it means randomized controlled trials or meta-analyses;^7,8,19^ in management, it may mean findings from cognitive psychology about how people assimilate information or what motivates them.^16,17^ In all these cases, knowledge is seen as unproblematically separable from the scientists who generate it and the practitioners who may use it (the ‘objectivist’ approach to knowledge).

The second assumption is that it is useful to conceptualise a ‘know–do gap’ between scientific facts and practice (whether in the clinical encounter, the management of staff or around the policy-making table). This implies that knowledge and practice can be cleanly separated both empirically and analytically. The third assumption is that practice consists more or less of a series of rational decisions on which scientific research findings can be brought to bear. These assumptions are widely held within the medical field, but as we argue below, they are also widely questioned by scholars outside this field.

## Alternative metaphors and models

The notion of knowledge as objective, context-free scientific facts which need to be ‘translated’ (summarized, packaged, prioritized, and presented in a form understandable and useable by practitioners) competes in the wider literature with a number of other conceptualisations of what knowledge *is* and how it is circulated and used (Table 1). For Aristotle, knowledge included not only *episteme* (facts) but also *techne* (skill) and *phronesis* (a form of practical wisdom). As Table 1 shows, many later philosophers have emphasized the importance of tacit knowledge (‘knowing how’ knowledge which is difficult to write down or transmit, such as speaking a language or riding a bicycle) and how this is built from experience, shared across communities and linked to action in context (‘constructivist’ and/or ‘performative’ approaches to knowledge).

**Table 1:** Different metaphors and models for knowledge, how it spreads and its relationship with practice

Discipline/tradition (with examples of key scholars)	Metaphor or shorthand description for knowledge	Metaphor or description for spread and distribution of knowledge	Implied link between knowledge and practice
*Perspectives consistent with ‘knowledge translation’*			
Clinical science	Research discoveries (laboratory science)	T1 knowledge transmission	*In vitro* discoveries are tested *in vivo* to generate clinical applications
Clinical epidemiology /evidence-based medicine	Research evidence (e.g. clinical practice guidelines)	T2 knowledge dissemination/translation	‘Evidence-based practice/policy’ = implementation of clinical research evidence
*Perspectives inconsistent with ‘knowledge translation’*			
Philosophy (Polanyi)^20^	Personal knowledge, embodied knowledge, tacit knowledge	Acquiring a way of engaging with the world	Knowledge is embodied, inseparable from the knower and contiguous with practice
Nichomachean ethics (Aristotle) and narrative medicine (Montgomery)^21^	Practical reason	Accumulation of experience under the supervision of wise and good teachers, reflection on practice, often transmitted as ‘stories’	*Praxis* is the ability to make wise, practical, ethical judgements (‘what best to do in this case’)
Philosophy (Wittgenstein)^36^ and ethnomethodology (Garfinkel)^37^	‘Language games’: the unwritten rules that members of a social group follow as they go about their everyday practices	Learning a set of rules (not by codification but by recognizing ‘family resemblances’ between different situations and contexts of action and acting them out)	Knowledge is a set of dispositions that people acquire and promulgate within a community, and which confer the ability to speak and act appropriately in a social situation
Cultural sociology (Bourdieu)^38^	Cultural capital, ‘knowing how’ rather than ‘knowing that’	Cultural and social [re]production through people's interactions	Knowledge is the socially acquired capacity or tendency of a person to act appropriately in given circumstances
Organizational sociology (Weick, Brown and Duguid)^27,39^	Individual: ‘sticky’ knowledge (cannot easily be passed on), ‘knowing the ropes’. Collective: shared representations, institutional logics, routines	Accumulation of experience, reflection on practice, informal storytelling (‘office gossip’), following routines	Knowledge is the ability to exercise judgement within a particular field of practice. It involves (a) the ability to draw distinctions and (b) connection with a collectively generated and shared domain of practice
Communities of practice (Lave and Wenger)^24^	Knowledge as socially shared practices, linked to membership and identity	Apprenticeship, social learning, legitimate peripheral participation (learning by ‘lurking’ in the community of practice)	Knowledge is contiguous with practice
Management studies/resource-based view of the firm (Nonaka)^40^	Knowledge (especially tacit knowledge) is a commodity or resource to be managed and thus a key contributor to profitability	The ‘knowledge creation cycle’ (socialisation, externalisation e.g. through storytelling, combination with other knowledge and internalisation)	Knowledge in an organization takes many forms, one of which is embodied in practice
Interdisciplinary perspective on healthcare (Davies)^29^	Diverse: research evidence plus tacit knowledge plus local knowledge, linked in a messy way	Knowledge interaction (‘messy engagement of multiple players with diverse sources of knowledge’) and knowledge intermediation (‘managed processes by which knowledge interaction can be promoted’)	Dynamically linked in a somewhat messy (but ultimately productive) way
Engaged scholarship (Van de Ven)^31^	What emerges when researchers and practitioners collaborate to address a practical problem	Co-production	Knowledge emerges from collaborative practice
Eclectic synthesis of all the above (Gabbay and le May)^25^	Mindlines (individually embodied, collectively reinforced, largely tacit guidelines)	The knowledge of research evidence is transformed and internalised through interaction with patients, reflection on practice, and exchange of stories with trusted colleagues (communities of practice)	Knowledge, practice and context are inseparable. An individual's mindline is one person's mental embodiment of their ‘knowledge-in-practice-in-context’, mediated through collective mindlines, so that they become ‘contextually adroit’

Wittgenstein, for example, rejected the idea that words have any fixed meaning outside the context in which they are used. The words ‘I do’ mean different things when responding to a question about one's lifestyle by a healthcare assistant, ticking an online box to indicate that one understands the terms and conditions of a sale, or getting married. Knowledge, Wittgenstein argued, is more about the subtleties of such ‘language games’ than about accumulating facts devoid of context. To understand a language game is to understand the wider structures in society such as norms, values, rituals, professional expectations, legal frameworks, economic and political constraints and how these are interpreted by particular groups in particular settings.

Some philosophers of science argue that the situated (local, context-dependent) nature of knowledge holds not only in the ‘soft’ social sciences but also in ‘hard’ physical and laboratory science. Polanyi, for example, argued that what seems like objective science progresses largely through the commitments, motivations and judgements of individuals (not least the creative decision of which research question to ask and what kind of methods best answer it).^20^ Knowledge is not merely derived from our senses and from the instruments we use to collect data; the discovery of one ‘scientific fact’ does not make the next experiment self-evident. Rather, facts always require a tacit awareness in which they can be framed (placed in context), interpreted (given meaning and value) and linked to further questions. Particular (invariably, powerful) social groups set research priorities, allocate funding, define what counts as ‘important’ questions, classify certain types of research as having greater or lesser value, and control the publication and distribution of scientific findings. To the extent that even ‘hard’ science is socially constructed, knowledge translation cannot be viewed as a politically neutral exercise in the transmission of facts.

## Knowledge in the clinical encounter

Clinical encounters are more than a collection of decisions: they are complex social accomplishments. The pop-up prompt ‘offer Chlamydia screening’ which appears on a general practitioner's computer screen during a consultation with a young person aged 15–25 is derived from an evidence-based guideline. Nevertheless, there is a balance to be struck between (on the one hand) the letter of the guideline and its underpinning evidence base and (on the other hand) the unintended consequences that may be generated by following it. Non-adherence to the Chlamydia guideline here is not explained by a simple ‘know–do gap’. Rather, the doctor must combine *both* relevant research evidence (such as the ‘number needed to screen’ – the number of young people that must be cold-questioned about their sex lives to prevent one case of infertility in the future) – *and* tacit knowledge of the wider clinical and social situation.

Montgomery has analysed such judgements in her book *How Doctors Think*.^21^ Drawing on Aristotle, she argues that despite its own emphatic claims to the contrary, medicine is not a science at all – and nor, incidentally, is it an art. Medicine is a *practice* – specifically, an uncertain, paradox-laden, judgement-dependent, science-using, technology-supported practice. As such, and despite all the scientific knowledge which informs it, medicine is comparable to the practice of law or making of ethical judgements. In every case, the practitioner must reason not from the general to the particular but from the particular to the general – abduction rather than deduction. The question facing every practitioner, every time they encounter a case, is: ‘What is it best to do, for *this* individual, at *this* time, given *these* particular circumstances?’ The skilled practice of medicine is not merely about knowing a set of abstracted rules and recommendations but about deciding *which* of many competing rules is most relevant. Faced with a 75-year-old with a high cholesterol level, should I follow the guideline which tells me to prescribe a statin – or the one which tells me to avoid polypharmacy in older people?

The ‘knowledge translation’ metaphor places such situated judgements (Aristotle's *phronesis* – the ability to apply general rules to particular situations) beyond the analytic frame.^21^ Yet phronesis is why, as the Dreyfus brothers observed, experts reason differently from novices and humans reason differently from computers.^22^ The ‘know–do gap’ will never be fully bridged by ‘evidence-based actionable messages’ or more refined combinations of sticks and carrots to ‘incentivize’ the use of research evidence in clinical encounters. Rather, the key to building a closer link between knowledge and practice will occur at least partly via what Kemmis (writing in the education literature) calls ‘personal praxis’^23^– the reflexive consideration, individually and collectively, of how one has performed (or should perform) in particular cases and situations. This concept is closely related to Lave and Wenger's notion of community of practice, in which the acquisition of ‘personal praxis’ goes hand in hand with the development of identity and participation in a social group.^24^

Gabbay and le May have taken this argument further based on their ethnographic study of the ways that general practitioners use what they call ‘knowledge-in-practice-in-context’. During their observations they never saw a clinician consult a guideline to help make a decision during a clinical encounter.^25^ The clinicians drew instead on complex and flexible internalized guidelines – ‘mindlines’ – which incorporate a wealth of different kinds of knowledge, explicit and tacit, general and specific, acquired over a lifetime of learning, reading and experience. Mindlines are continually being adjusted partly by grazing on written sources but mainly by reflecting on experience during discussions with colleagues and opinion leaders, including sharing stories of how they managed real cases.

## Knowledge in organization and management

In her 2006 Presidential address to the Academy of Management, Deborah Rousseau proposed ‘evidence-based management’, comprising (a) learning about cause-effect connections in professional practice; (b) isolating the variations that measurably affect desired outcomes; (c) creating a culture of evidence-based decision-making and research participation; (d) using information-sharing communities to reduce over-use, under-use and misuse of specific practices; (e) building decision-support systems to promote practices the evidence validates, along with techniques and artefacts that make the decision easier to execute (e.g. protocols, checklists); (f) promoting access to knowledge at individual and organizational level. The message that particular tools and techniques can, through systematic research, be shown to be effective or ineffective and thence either promoted into, or discouraged out of, organizational settings – is the founding assumption behind the National Institute of Health Research Service Delivery and Organisation Programme (see http://www.sdo.nihr.ac.uk/), whose director co-authored a paper on evidence-based healthcare management back in 2001.^16^

Critical voices remain unconvinced. Learmouth, for example, has argued that while efforts to improve on the inconsistent and *ad hoc* approaches that are rife in management practice are laudable, the underpinning assumptions of evidence-based management are flawed – e.g. that the goal of management is to increase profit.^26^ ‘Facts’ in evidence-based management (as in evidence-based medicine) are depicted as value-free, waiting to be collected through research and serving the interests of no specific group. The counter-argument is that ‘in a social science like organization studies, “evidence” is never just there, waiting for the researcher to find. Rather, it is always necessary to construct it in some way – a process that is inherently ideological and always contestable – not merely a technical, “scientific” task’ (page 95).^26^ Management is by nature a pluralist field; different theoretical (and ideological) approaches may be relevant in different contexts. It follows that ‘evidence-based management’ will necessarily serve the interests of dominant elites (e.g. top management), because it will be they who define the questions and produce the standards by which ‘best’ evidence is judged.

Critical scholars in organization and management have sought to promote what they call a social practice view of knowledge – that is, that the key challenge is not to accumulate and distribute placeless, timeless, value-free ‘facts’ about management practice but (in relation to *particular* challenges now and in the future) to identify, manage and mobilize the many different types of knowledge generated by the diverse communities of practice which exist within and across organizations.^27^ If the question is ‘how should we work towards our organizational mission?’, for example, the answer will not be found in some abstracted manual of ‘best strategies’ but in the shrewd and careful analysis of information on the case in hand.

## Knowledge in policy-making

The argument that research findings cannot be ‘transferred’ in a simple, linear way into policy has been made previously;^28,29^ we summarize it briefly here. Policymakers have many legitimate goals other than clinical effectiveness (e.g. terms and conditions of public employees; balancing the books; accounting to parliament); scientific evidence is often ambiguous, incomplete, partisan and open to multiple interpretations; tacit and local knowledge may be relevant to policy decisions; it may be practically impossible to change policy in a particular ‘evidence-based’ direction; and research findings may serve to challenge general ideologies and assumptions as much as to inform specific decisions. Furthermore, policy-making may be best viewed not as a rational exercise in decision science (for which clear, actionable evidence on ‘what works’ would be the perfect substrate) but as a process of argumentation to decide what is right and reasonable (e.g. given limited resource, should we fund a cardiac rehabilitation programme, an outreach service for acute psychosis or an expansion in infertility services?); in such circumstances, research evidence may be used instrumentally and rhetorically to back up particular value-based positions. This occurs particularly when there is ‘high issue polarisation’ – that is, disagreement among stakeholders about what the significant problems are and how they might be addressed.^5^

These (and other) complexities help explain the emergence of a relatively new taxonomy of knowledge: ‘Mode 1’ (conventional scientific research, driven by curiosity and dispassionate inquiry, which produces evidence that is taken up and applied – or not – by decision-makers who had no influence on its focus or approach) and ‘Mode 2’ (research which emerges from active, two-way partnerships between researchers, decision-makers, funders, industry and other stakeholders). Whereas Mode 1 knowledge needs to be ‘translated’ in order to be applied, the research which generates Mode 2 knowledge is considered to be part of the context of application from the outset.^9,30,31^ Some say that the term ‘knowledge translation’, when used correctly, implies the development of partnerships and a two-way flow of knowledge even in Mode 1 research;^2,18^ others distinguish this bidirectional but still linear flow (in which research findings remain privileged over other forms of knowledge), from the term ‘knowledge exchange’ which depicts the non-linear, multi-stakeholder and interactive dialogue on which successful, policy-relevant research is built (and in which practitioner knowledge, industry knowledge and so on are afforded equal status with research findings).^5,10^

The generation of Mode 2 knowledge may be aligned with either the political left (as in participatory action research, power-sharing partnerships with patient groups and so on^32^) or the political right (as in the strengthening of links between academia and the biotech industry^33,34^). Between these two extremes, it is invariably a complex, non-linear and locally contingent process, for which ‘terms such as knowledge transfer (and its subordinate sibling, knowledge translation) misrepresent the tasks that they seek to support’ (page 188).^29^ Davies has argued for the term ‘knowledge interaction’ to convey the notion that the coming-together of stakeholders to generate and share knowledge may be conflict-ridden.

Nowotny and others have argued that the growing interest in, and credibility of, Mode 2 knowledge represents far more than a recognition that knowledge transfer should be ‘bidirectional’ (in the sense that researchers might ascertain, and seek to fill, policymakers' ‘knowledge gaps’).^33,34^ Rather, they suggest, Mode 2 research represents a fundamental shift in the way knowledge is produced. While it appears to fix the problem of ivory tower academics ploughing their own furrow oblivious to the problems of society, it engenders new and potentially sinister forms of symbiosis between government, industry and science.^35^ Research is increasingly a policy issue, its priorities set at national level with overt government influence: it must be programmatic, collaborative, relevant, cost-effective and generate ‘innovations’. Lamentably, pursuit of knowledge as a public good (or for some other, perhaps critical, purpose) is increasingly discouraged.^9^

## Conclusion

Conceptualising the generation, circulation and sharing of knowledge as ‘translation’ will inadvertently close our minds to alternative framings which could add to the illumination and analysis of this complex field. We propose that the terms of engagement for debate be redrawn – and specifically, that the term ‘knowledge translation’ be joined by a wider menu of metaphors and models such as ‘phronesis’ (practical wisdom), ‘mindlines’, ‘knowledge intermediation’ and even ‘language games’. Furthermore, we suggest that the research agenda be renamed and broadened to address the following issues:

First, research is needed on case-based reasoning – that is, on how doctors and other practitioners balance the generic recommendation of a guideline or protocol against the particularities of a case in the here-and-now (including weighing up competing recommendations), especially but not exclusively when such recommendations are inscribed in technology as templates or pop-up prompts. We suggest that research designs such as ethnography and the detailed micro-analysis of transcripts of consultations might be particularly suitable here. Such an approach would be equally suited to studying the situated practices of managers, administrators and others involved in the organization and delivery of care.

Second, we should systematically research the development and activity of communities of practice with a focus on ‘mindlines’.^25^ The emergence of online communities of practice in facilitated online forums creates new opportunities for researching the collective conversations and deliberations through which mindlines evolve. Again, research into this collective dimension of knowledge is likely to involve the detailed micro-analysis of talk and text.

Third, as Crilly and colleagues concluded in their systematic review of knowledge management research,^6^ much might be gained by applying research approaches and techniques from critical management studies (see Learmouth above) to the study of the link between power and knowledge in the healthcare field. The technique of discourse analysis might be used, for example, to make explicit the process by which certain types and sources of knowledge become defined as ‘best evidence’ at the expense of others and how semi-automated metrics such as league tables and journal impact factors reinforce and reproduce particular versions of the ‘evidence hierarchy’. The influence of the pharmaceutical industry, medical device manufacturers, commercial software companies, management consultants, research leaders and political and third-sector lobbyists in defining what counts as research knowledge and mobilising resources to generate and distribute it should be systematically and critically studied.

Fourth, while there is already much published research on how multiple forms and sources of knowledge come together, sometimes harmoniously but more commonly with some discord, in the policy-making process and other macro-level interactions (Huw Davies' ‘knowledge interaction’), there is as yet very little research on what Davies called ‘knowledge intermediation’ – the ways in which such interaction might be productively facilitated and supported. The sinister potential of Mode 2 knowledge partnerships to build hidden biases into the design of research programmes, for example, may be attenuated in part by independent facilitation in which such dangers are made explicit and addressed as part of the governance process. In-depth organizational case study is likely to be the study design of choice here.

Finally, we propose research into strategic-level approaches to the cycle of developing, implementing and revising clinical guidelines in a way that recognises and captures practical wisdom and case knowledge. In other words, we should not only research how mindlines emerge and evolve but also how we might systematically facilitate their emergence and evolution. New technologies such as secure social-networking sites are an important part of this picture, but the research agenda here should not be overly technology-focused since it must also consider governance, support from professional bodies and embedding within the wider health system.

In sum, we believe that while the generic concept of knowledge translation is, broadly, a ‘good thing’, the precise term has outlived its usefulness. Before the assumptions behind it become fully entrenched, we should broaden our conceptualisation of knowledge and extend what we consider to be good research in this field of practice.

## DECLARATIONS

### Competing interests

None declared

### Funding

None

### Ethical approval

Not applicable

### Guarantor

TG

### Contributorship

Both authors searched the literature and contributed references; TG wrote the paper with assistance from SW

### Acknowledgements

This summary article draws on previous work by others. The authors thank John Gabbay and Andréé le May for helpful comments on an earlier draft of this manuscript, and in particular for suggesting extensive improvements to Table 1, and Huw Davies for a prompt and helpful review of an earlier draft
